# Degradation of GaAs/AlGaAs Quantized Hall Resistors With Alloyed AuGe/Ni Contacts

**DOI:** 10.6028/jres.103.012

**Published:** 1998-04-01

**Authors:** Kevin C. Lee

**Affiliations:** National Institute of Standards and Technology, Gaithersburg, MD 20899-0001

**Keywords:** alloyed contacts, contact degradation, GaAs, gold-germanium-nickel, ohmic contacts, passivation, quantized Hall resistor, quantum Hall effect, 2-dimensional electron gas

## Abstract

Careful testing over a period of 6 years of a number of GaAs/AlGaAs quantized Hall resistors (QHR) made with alloyed AuGe/Ni contacts, both with and without passivating silicon nitride coatings, has resulted in the identification of important mechanisms responsible for degradation in the performance of the devices as resistance standards. Covering the contacts with a film, such as a low-temperature silicon nitride, that is impervious to humidity and other contaminants in the atmosphere prevents the contacts from degrading. The devices coated with silicon nitride used in this study, however, showed the effects of a conducting path in parallel with the 2-dimensional electron gas (2-DEG) at temperatures above 1.1 K which interferes with their use as resistance standards. Several possible causes of this parallel conduction are evaluated. On the basis of this work, two methods are proposed for protecting QHR devices with alloyed AuGe/Ni contacts from degradation: the heterostructure can be left unpassivated, but the alloyed contacts can be completely covered with a very thick (> 3 μm) coating of gold; or the GaAs cap layer can be carefully etched away after alloying the contacts and prior to depositing a passivating silicon nitride coating over the entire sample. Of the two, the latter is more challenging to effect, but preferable because both the contacts and the heterostructure are protected from corrosion and oxidation.

## 1. Introduction

Quantized Hall resistors (QHRs) made with alloyed AuGe/Ni ohmic contacts to GaAs/AlGaAs heterostruc-tures are quite widely used as resistance standards by many national standards laboratories [1, 2]. These devices are repeatedly cooled and warmed between room temperature and temperatures below 1.4 K over periods of many years. Degradation or failure of the devices during cooling or use is costly, for the laboratory’s calibration schedule is delayed, both liquid helium and staff time are lost while the device is replaced or repaired, and a lengthy testing procedure must be performed to certify a new or repaired device as a resistance standard [3]. It is therefore of great importance that the devices used as resistance standards be as reliable and resistant to degradation as possible. Degradation of the devices can result from processing steps used to mount the devices in packages, but can also occur over a period of many years as humidity and atmospheric contaminants corrode or oxidize the contacts and the heterostructure.

In previous work [4] it was shown that bonding wires to the contact pads directly over the heterostructure results in the formation of electrically active defects in the fragile heterostructure beneath the contacts, which increases the contact resistances and degrades the performance of the device. This degradation can be eliminated by depositing bonding pads that extend over both the contacts on the heterostructure and the semi-insulating substrate to permit wires to be bonded over the substrate. Any damage to the substrate caused by the high pressures created during the bonding process will then not affect the sensitive ohmic contact to the heterostructure.

The work reported in this paper concerns the causes of long-term degradation in QHR devices that occurs over a period of many years. Quantized Hall resistance devices with alloyed AuGe/Ni ohmic contacts on GaAs/AlGaAs heterostructures both with and without passivating silicon nitride coatings were studied. Enlarged bonding pads were deposited over the alloyed contacts and wires were bonded to the pads over the substrate, so the sensitive alloyed contacts were not exposed to any mechanical stresses. When the samples were tested, it was found that those with silicon nitride coatings had very low contact resistances and were of very high quality, but the minima in the voltages measured between probes on the same side of the Hall device (*V*_x_) under the conditions required to observe the quantum Hall effect (QHE) did not vanish at temperatures above 1.1 K as is required for use as resistance standards [3]. While the minima in *V_x_* did vanish for samples that were not coated with silicon nitride and which had been stored in plastic petri dishes for over 6 years in an unregulated laboratory environment, these samples were found to have higher contact resistances and somewhat more nonuniform electron concentrations than the coated samples. The higher contact resistances are attributed to the fact that the AuGe/Ni contacts on the uncoated samples were exposed directly to corrosive compounds in the laboratory atmosphere, where the temperature and humidity varied over a wide range (18 °C to 30 °C, 10 % to 70 % relative humidity). Corrosion of unprotected metal contacts under these conditions has been reported in the literature [5, 6, 7]. The higher nonuniformity of the electron concentration in the samples without a silicon nitride coating is attributed to nonuniform oxidation of the exposed top surface of the heterostructure.

These observations indicate that it is necessary to protect the AuGe/Ni ohmic contacts on GaAs/AlGaAs QHE devices from the atmosphere in order to ensure their long-term reliability. Two techniques for protecting the contacts are proposed as a result of this work. The simplest is to cover the contacts completely with a coating of gold greater than 3 mm in thickness. Such a coating has been shown to prevent corrosion in metal contacts [5]. The other is to cover the samples with a silicon nitride layer deposited using a low temperature chemical vapor deposition (LTCVD) technique, as was done with the passivated samples used in this study. The nonzero minima in *V_x_* measured on these passivated samples do not appear to be due to current flowing through the nitride, as has previously been supposed [1], but is most likely due to current flowing in the degenerately doped GaAs cap layer. If this cap layer is grown without donor impurities or is etched off after alloying the contacts and prior to depositing the nitride, this conduction should be eliminated, and the *V_x_* minima should vanish as is required of standards-quality QHR devices. The nitride coating should then protect both the contacts and the heterostructure from corrosion and degradation.

Section 2 of this paper gives a brief description of the samples used in this study. The details of the procedure used to mount the samples for quantum Hall effect measurements are given in Sec. 3. A summary of the results of QHE tests on both the samples covered with a passivating silicon nitride coating and those without is given in Sec. 4. In Sec. 5 the causes of degradation of unprotected AuGe/Ni contacts are discussed, and in Sec. 6 two methods are proposed for preventing this degradation from occurring.

## 2. Origin and Design of the QHE Devices

**Fig. 1:**
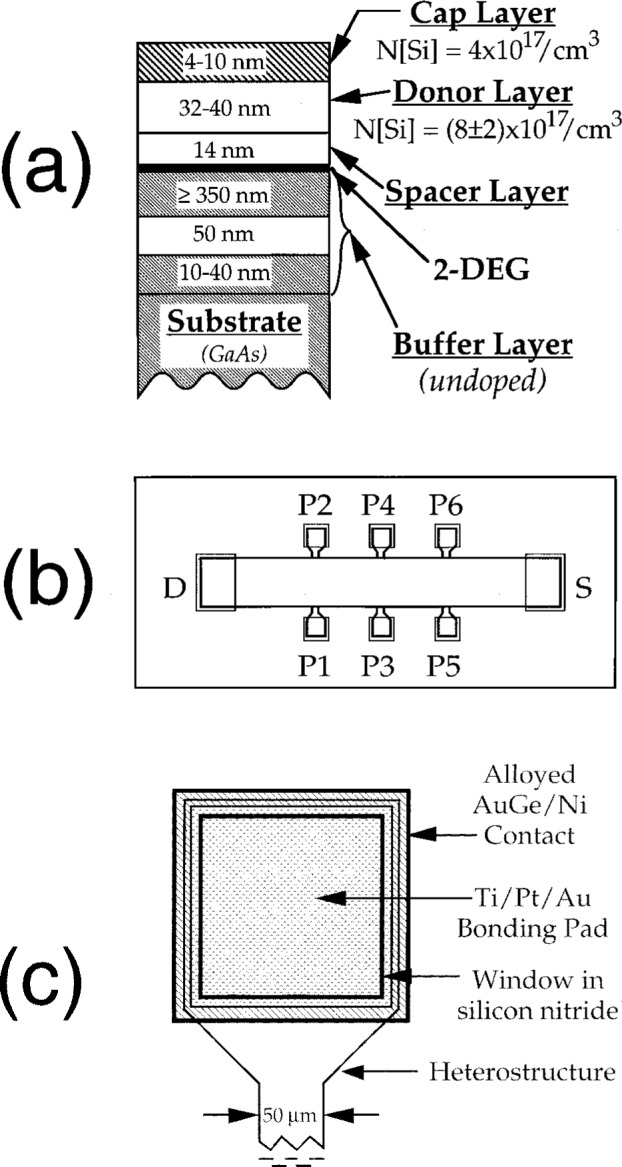
Design of LEP samples. (a) Schematic cross section of the heterostructure from which the EUROMET samples were made (from Ref. [1]) (b) Scale drawing of the mask used by LEP to make the EUROMET samples. (c) Detail of the region containing the ohmic contact on a potential pad on a sample coated with silicon nitride.

In 1990, the EUROMET consortium of European national standards laboratories, in conjunction with the Bureau International des Poids et Measures (BIPM) in France, the National Institute of Standards and Technology (NIST) in the USA, and the National Research Council (NRC) in Canada, let a contract with the Limeil GaAs Foundry of the Laboratoires d’Electronique Philips (LEP)^1^ in France to produce quantized Hall resistance devices (see Ref. [1]). LEP grew a GaAs/Al-GaAs heterostructure, a schematic cross-section of which is shown in Fig. 1a, using the technique of Metal-Organic Vapor Phase Epitaxy (MOVPE). The top layer of the heterostructure is a GaAs cap layer doped with silicon; below it are a donor layer and spacer layer of Al_0.28_Ga_0.72_As. The donor layer is doped with silicon atoms, but the spacer is not. The “buffer layer” is composed of two layers of GaAs separated by a layer of Al_0.1_Ga_0.9_As, all undoped. The location of the 2-dimensional electron gas (2-DEG) responsible for the quantum Hall effect is shown by the black line labeled “2-DEG.” The Al_0.1_Ga_0.9_As layer in the buffer layer helps isolate the channel in which the 2-DEG resides from defects in the substrate, and is also intended to minimize the injection of high velocity electrons (also called “hot electrons”) from the 2-DEG into the buffer layer and substrate, a problem that is much more severe with high electron mobility transistors (made using this same design of heterostructure) than with QHE devices [8].

The EUROMET committee provided LEP with a pattern for a Hall bridge and ohmic contacts, shown in Fig. 1b, based on designs used at European standards laboratories. The length of the Hall bar is approximately 2.7 mm and its width is about 0.4 mm. The Limeil Foundry etched the Hall bar pattern into the heterostructure, and alloyed gold-germanium-nickel contacts to the samples. Ti/Pt/Au bonding pads were deposited on top of the AuGe/Ni pads (see Fig. 1c). The grey squares in Fig. 1b indicate the locations of the alloyed gold-germanium nickel contacts. The labels D and S in the figure identify, respectively, the drain and source contacts through which current flows, and the pads labeled P1… P6 identify the potential probes. The heterostructure mesa beneath the potential pads is about 160 μm in width, and the pad neck is about 50 μm in width, as indicated in Fig. 1c. The AuGe/Ni alloyed contacts are 175 μm square, and extend about 7.5 μm beyond the edge of the heterostructure mesa. The Ti/Pt/Au bonding pads are 152 μm square, and were deposited over the AuGe/Ni alloyed contacts, entirely within the mesa: the edge of the Ti/Pt/Au contact is about 4 μm inside of the edge of the heterostructure mesa.

On half of the devices, a protective 165 nm thick silicon nitride coating was applied using low-temperature chemical vapor deposition (LTCVD). This nitride coating covers the entire sample, except for holes exposing all of the Ti/Pt/Au bonding pads with the exception of a 2.5 μm wide rim around the edge of the bonding pads, which rim lies under the nitride. The hole over each potential probe, shown in Fig. 1c, is 147 μm square.

These devices were given to the individual national standards laboratories to be mounted and tested. In the autumn of 1990, NIST received 30 devices without the nitride coating and 30 devices with the nitride coating. These devices were used in the work reported in this paper.

## 3. Procedure Used to Mount the Devices

In order to use these devices as resistance standards, they must be mounted in packages, typically 12-pin, nonmagnetic “headers,” that fit into sockets in the probe of a cryogenic system that can cool them to temperatures below 1.2 K in magnetic flux densities of between 5 T and 8 T required to observe the quantum Hall effect. In principle, the procedure for doing this is quite simple: the sample is attached to the package using epoxy, and wires are bonded between the pads on the sample and the header pins using standard wire bonding techniques. If one uses very soft, 12 μm diameter wire when bonding, this simple procedure can probably be used without harming the contacts, for the forces generated when such wire is bonded to pads on the GaAs/AlGaAs heterostructure are quite small. Such small diameter wire is, however, very fragile, and can easily be broken by gusts of helium gas or other stresses generated when the sample is cooled to cryogenic temperatures. Larger diameter wire, such as the 25 μm diameter wire used in this study, is much sturdier and can better withstand the stresses generated during cooling, but such wire requires the use of higher bonding forces which generate greater stresses on the semiconductor underneath the pad to which the wire is bonded. As discussed in an earlier paper [4], the heterostructure is extremely fragile, and quantized Hall resistance devices are extremely sensitive to the slightest damage in the contact region. Hence, the forces generated during bonding of 25 μm diameter wires create electrically active defects in the heterostructure underneath the bonding pad even when the lightest bonding pressures are used, resulting in a measurable increase in the resistance of the contacts.

To prevent this damage, large bonding pads, overlapping both the alloyed contact and a substantial part of the substrate, were deposited, and wires were bonded to these pads over the substrate, rather than over the heterostructure. Defects created in the substrate due to the high pressures required to bond wires to the bonding pads therefore do not affect the quality of the ohmic contact, because the substrate is semi-insulating and does not carry any current during normal operation of the device. All of the devices used in this study have been mounted using procedures based on this “enlarged bonding pad” principle. The next section (Sec. 3.1) discusses some of the challenges experienced with processes used in early trials, and Sec. 3.2 describes the final procedure used to mount the majority of the samples used in this study.

### 3.1. Principles of the Procedure

There are a number of ways in which this “enlarged bonding pad” principle can be implemented. All procedures must, however, ensure that:

- bonding pads are deposited over the alloyed contacts and make good electrical contact with them—there must be no organic contamination on the contacts prior to deposition of the bonding pads;
- the bonding pads are very adherent to the substrate and are thick enough so stresses created during bonding do not cause the bonding pads to tear away from the substrate;
- and the method used to define the bonding pads does not disturb the alloyed contacts.

**Fig. 2:**
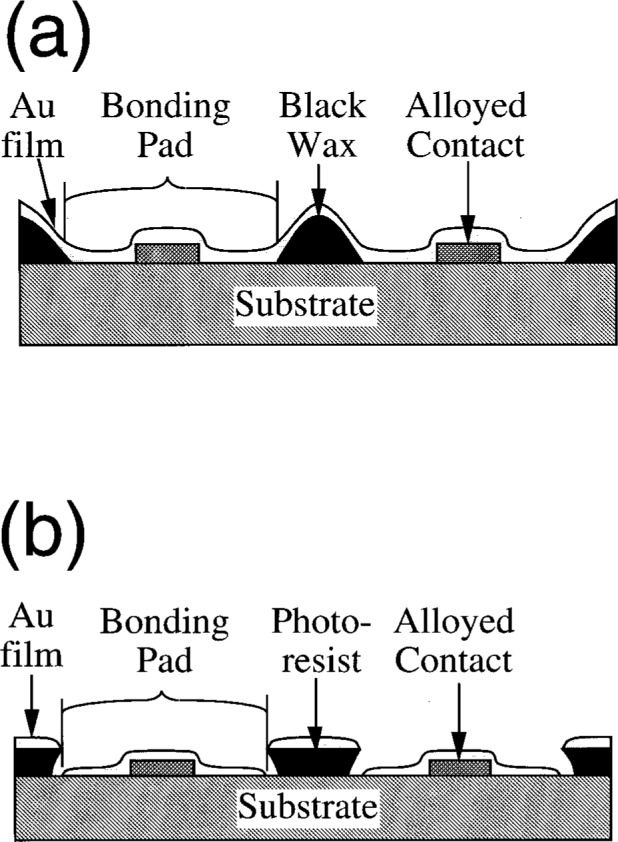
Illustration of two different methods of defining a pattern of enlarged bonding pads on the LEP samples: (a) Application of a black wax pattern results in a continuous metal film that covers both the sample and the wax mask. The metal between the bonding pads must be removed by vigorous agitation in an ultrasonic cleaner. (b) Application of photoresist results in a discontinuous metal film that can be simply lifted off by dissolving the resist in acetone, resulting in well-defined bonding pads without the need for vigorous ultrasonic agitation of the sample.

In early experiments, Apiezon W (black wax made by Apiezon products, London, UK) was applied to the regions of the sample to which the metal film that would form the bonding pad would *not* adhere, the metal was deposited, and the black wax was dissolved. Because the evaporated gold film continuously coated the sample as shown in Figure 2a, dissolution of the black wax in solvent did not separate the adjacent bonding pads. Vigorously agitating the sample in an ultrasonic cleaner successfully fragmented the free-standing film that had covered the black wax, thus resulting in well-defined bonding pads over each ohmic contact. Because the evaporated metal adhered very strongly to silicon nitride, this patterning technique worked well for the passivated samples. The metal film did not adhere as well to the GaAs substrate on the unpassivated samples, and the agitation required to fragment the free-standing gold film that had been deposited over the black wax also tended to remove the evaporated film from over the alloyed contacts, where it was supposed to remain.

This problem is readily solved by applying photoresist to the sample and using the “lift-off” procedure [9]. As shown in Fig. 2b, this results in a discontinuous metal film. When the photoresist is dissolved in acetone, the metal between the bonding pads is removed easily without the need for vigorous ultrasonic agitation. The metal film has to be fairly thick (a minimum of 0.3 μm and preferably thicker) in order that it be able to absorb enough of the bonding stresses to minimize damage to the substrate, and to prevent the stress at the metal-substrate interface from reaching a value at which the metal will shear and peel away from the substrate. In addition, for this procedure to work properly, the resist must be considerably thicker than the metal in order that the metal not form a continuous bridge over the resist. This requires the application of uniformly thick resist films greater than 1 μm in thickness to chips 0.2 mm thick with width and length of 1.1 mm and 2.9 mm, respectively, without the formation of a bead of resist around the edge of the chip (commonly referred to as an “edge bead”). The absence of the edge bead was particularly important, for the contact pads were less than 100 μm from the edge of the chip.

While this task is rather challenging, a technique for doing it reliably was developed. A depression with nearly the exact dimensions of the chip was made in a glass plate and the sample was affixed to this plate with a minute quantity of photoresist. The plate was then heated very slowly to drive the solvent out of the photoresist without causing the formation of bubbles that would force the sample up out of the depression in the plate. The surface of the sample was then precisely even with the top surface of the glass plate, so the resist film on the sample was quite uniform. This technique was successfully used to mount a few of the samples coated with a passivating nitride layer without significant degradation of the contacts. When attempts were made to mount unpassivated samples using this technique, however, the resistances of the contacts were quite noticeably increased, even at room temperature. The alloyed AuGe/Ni contacts on the unpassivated samples were directly exposed to all of the processing solutions, and it was found that they are degraded quite noticeably by exposures for periods of time as short as a few minutes to several different varieties of photoresist developer made by different manufacturers, as well as by photoresist remover.

In view of the extreme sensitivity of the exposed alloyed contacts on the unpassivated samples to corrosion, the bonding pads can only be applied by evaporating the metal through a “see through” mask, a thin metal foil with holes etched through it in the appropriate places, that is placed in proximity to the sample while the metal is evaporated. The sample can only be cleaned in inert solvents, such as xylenes, tricholorethylene, or acetone, for brief periods, with relatively little ultrasonic agitation.

### 3.2. Annotated Processing Sequence

The procedure used to mount the samples in this work is based upon the principles described in the previous section, namely:

1. The samples must only be cleaned in inert solvents with a minimum of agitation—the samples must *never* be exposed to any aqueous or caustic or corrosive solutions, even those that are not water-based;
2. Thick bonding pads, covering both the alloyed contact and the heterostructure, must be deposited through a “see-through” mask;
3. Exposure of the sample to temperatures of 200 °C or higher during bonding and curing of the epoxy should be minimized.

The processing steps in the procedure used in this work, hereafter referred to as the “optimized procedure” are now described.

1. Teflon FEP (Fluorinated Ethylene Propylene) beakers were used to clean the samples. The beakers were first cleaned by boiling in them a solution of 1 part 98 % H_2_SO_4_ to 1 part 30 % H_2_O_2_ to between 3 and 5 parts by volume of deionized water (with resistivity of 18 MΩ cm) for at least 10 min, followed by a thorough rinse with filtered, deionized water. Note that this process ensures that the beakers are clean before the samples are placed in them. This step is necessary because not only are the AuGe/Ni contacts very sensitive to corrosion in strong cleaning solutions such as wafer detergents, but GaAs itself is oxidized in almost all aqueous cleaning solutions.^2^ Thus, the only agents that can be used to clean the samples are solvents, which are not particularly strong cleaning agents, so care must be taken to ensure that the samples never get very dirty.
2. The chip with the QHR device and a glass carrier plate^3^ with evaporated gold bonding pads (made separately) are placed in separate beakers and cleaned by first heating in xylenes^4^ for 10 min, and then agitating in an ultrasonic cleaner for 5 min. The xylenes are then decanted, and the procedure repeated with trichloroethylene, acetone, and finally methanol. The sample was then boiled briefly in acetone, the acetone decanted, and the samples blown dry with filtered dry nitrogen.
3. A weight of Au wire sufficient to produce a coating on the sample of at least 340 nm (preferably at least 0.5 μm thick) is cut, wound into a small ball about 5 mm in diameter, and cleaned by etching in a solution of 1 part 98 % H_2_SO_4_ to 1 part 30 % H_2_O_2_ to 5 parts by volume of water. This solution is first heated on a hotplate to between 80 °C and 100 °C for about 5 min, and then placed in an ultrasonic cleaner, where it is agitated for between 10 min and 15 min. the wire is then boiled in methanol and blown dry. In this work, an NRC 3114 Vacuum Coater with a source-to-sample distance of about 17 cm was used. In this evaporator a 1 g charge of gold produces a gold coating about 0.33 μm thick.
4. A glass microscope slide is heated to 90 8C on a small hotplate and the sample is mounted on it with black wax. The slide is then placed in a specially designed device, in which the “see-through” mask, made from a 25 μm thick brass foil, is clamped between two support plates, and the sample is held in close proximity to, but not in direct contact with, the mask.
5. The sample is placed in the evaporator. The gold wire is heated briefly with a torch until it glows reddish-orange, and then placed on a 50 mm long, 250 μm thick molybdenum strip with a thin alumina coating. A 50 mm long chromium-plated tungsten rod is also heated to red heat with a torch, and is then placed in the second filament bay in the evaporator. Heating the evaporation charges immediately prior to installing them in the evaporator removes any residual organic contamination, drives off adsorbed water vapor or solvent residues, and minimizes the amount of gas evolved from them when they are heated in vacuum prior to coating the samples.
6. The evaporator is evacuated for about 1 h until a base pressure of between 20 μPa and 65 μPa (0.15 μTorr to 0.49 μTorr) is reached. The liquid nitrogen trap between the diffusion pump and the chamber is then filled. Cooling water is supplied to the current feedthroughs to prevent them and the baseplate from heating up during the evaporation. Separate power supplies are used to supply current to the Cr and Au filaments, and both filaments are heated simultaneously. The sample is protected behind an aluminum plate while the filaments are being heated to evaporation temperature. Chromium is deposited on the sample first, typically for 2 min to 4 min with a power of 154 VA, resulting in Cr films on the sample that are between 17 nm and 35 nm thick. The sample is then rotated until it is over the gold filament, and deposition of the gold is commenced immediately. The gold charge is evaporated to completion, typically for 15 min to 30 min with a filament power between 350 VA and 400 VA. The final thickness of the Au coating is determined by the weight of the gold charge as described in paragraph 3 above, and was typically between 320 nm and 340 nm in these experiments.
  Gold will not adhere to most substrates, including GaAs, without the presence of some other element, usually a transition metal like chromium, titanium, or tungsten. Chromium was used in these experiments because of the ready commercial availability of conveniently used chromium-plated tungsten rod evaporation sources.
  Experience has shown that even with a low pressure in the evaporation chamber and a liquid nitrogen trap between the diffusion pump and the chamber, the strength with which the Au film adheres to the chromium layer decreases markedly as the time between the end of the chromium deposition and start of the gold deposition increases. By heating both filaments to the evaporation temperature nearly simultaneously so that the deposition of the gold film could be started immediately after deposition of the chromium, the adhesion of the bonding pads to the sample was maximized.
7. After the bonding pads have been deposited, the sample is removed from the evaporator and cleaned by heating in xylenes for about 5 min, and agitating it in an ultrasonic cleaner for another 5 min. The xylenes are then decanted, the sample boiled in trichloroethylene for a few minutes, and then blown dry with filtered dry nitrogen.
8. A small quantity of epoxy is then applied to the glass carrier plate and the sample placed in the epoxy. The glass plate is then placed on a hotplate at 165 °C for 5 min to cure the epoxy. The epoxy must meet rather demanding requirements: it must be strongly adherent at temperatures of about 200 °C to ensure that it holds the sample firmly during wire bonding in order to minimize the damage to the substrate that would result from movements of the sample during bonding, and yet must also not crack or fail at cryogenic temperatures of 1.2 K or less at which the quantized Hall resistors are operated.
  Trial was made of a number of different commercial epoxies. Conductive epoxies from two different manufacturers, one a single component epoxy and the other a two-component epoxy, were tried in several experiments. The single component, silver-filled conductive epoxy was found to adhere well at all temperatures and was extremely convenient to use, but its high conductivity meant that extreme care had to be exercised to ensure that none of the epoxy touched any of the bonding pads. The two-component conductive epoxy from the second manufacturer suffered from the same problem and furthermore tended to be crumbly at the bonding temperature of 200 °C, and so was judged not suitable for this application.
  In most of these experiments, a two-component, nonconducting epoxy, EPOTEK H70E (made by Epoxy Technology, Billerica, MA, USA) was used. It was found that if care was taken to prepare the epoxy using equal *weights* of the two components [10], the epoxy remained strongly adherent to the sample over the entire temperature range from 200 °C to less than 1.2 K. Even though the epoxy is insulating, care has to be taken to ensure that not too much epoxy is used so that neither the epoxy nor its residues contaminate the tops of the bonding pads during curing, as this makes it difficult to bond wires to the pads [11].
9. The carrier plate was then attached to a clean TO-8 header, also with epoxy, and gold wires with a diameter of 25 μm, a tensile strength of 5.9 cN (centinewton), and “4 % elongation” were bonded between the bonding pads on the sample and the pads on the carrier plate, and then between the pads on the carrier plate and the head pins. The sample was maintained at 200 °C during bonding. The bonding tool was pressed against the sample and ultrasonic power (approximately 250 mW) was applied for about 100 ms. The tool was pressed onto the pads on the sample with a force of between 25 cN and 30 cN; when bonding to the header pins and the pads on the glass carrier plate, bonding forces between 39 cN and 49 cN were used. Typically, one wire was bonded to each potential pad, and two wires were bonded to each source and drain contact pad, each to a different header pin (there were therefore two header pins connected to the source and two to the drain, so that the sample could continue to be used even if one of the pair of wires got broken). All the wires were bonded within a period of 30 min to 45 min.

This optimized procedure was used to mount most of the samples used in this study. Figure 3 shows a photograph of a typical sample after the completion of this procedure. A “streamlined procedure” was developed, in which:

1. the sample was affixed to the glass carrier plate with epoxy;
2. the carrier plate with attached sample was then cleaned in solvents (step 2 above);
3. the sample was baked for about 1 hour at 200 °C to desorb water vapor, and placed in the mask holder,
4. the chromium and gold films were evaporated; and,
5. the glass carrier plate was immediately mounted in the header and the wires bonded without post-evaporation cleaning.

This procedure was tested on one sample (designated “E8”), but the resistances of the contacts on this sample were higher than those of other samples mounted using the normal procedure described above. While the contacts on this one sample may have been of poorer quality before processing than those mounted with the optimized procedure, it is possible that some steps or steps in the streamlined procedure degraded the contacts. If this were the case, the most likely causes for the degraded contacts would be residues from the epoxy (applied in the first step) not removed by cleaning in solvents (second step) which may have contaminated the AuGe/Ni contacts resulting in poor electrical contact between the evaporating bonding pads and the alloyed contacts. The high-temperature baking step (step 3) may have led to some change in the composition of the contacts that may have increased their resistances. For these reasons, the “streamlined procedure” was not used to mount more than the single test sample.

## 4. Results

The optimized procedure described in the previous section was used to mount three LEP samples without passivating silicon nitride coatings with serial numbers E5, E6, and E7. One unpassivated LEP sample (E8) was mounted using the “streamlined procedure” described above. Two passivated samples (serial numbers E5C and E7C) had been mounted in 1993 using a technique identical to the optimized procedure, except that black wax was used to define the bonding pads, as described in Sec. 2, rather than a “see-through” mask. Tests on sample E7C were reported in Ref. [4] in the section entitled “Enlarging Bonding Pads.” These samples were compared with one coated sample (E2C) and one uncoated sample (E1) that were mounted in headers and tested in 1990 shortly after the samples were received at NIST. Wires were attached to the contact pads on these samples by melting small beads of indium onto the gold pads directly over the heterostructure and pressing the gold wires into the beads. The results of the tests on these samples were reported in the “Soldering” section of Ref. [4].

All samples were tested under the conditions required to observe the quantum Hall effect. Because the superconducting solenoid used in this experiment was limited to a maximum magnetic flux density of 8 T, only the *i* = 4 plateaus were examined in this work. Under these conditions, the sample resistance is *V*_H_/*I* = *R*_K_/4 = *h*/4*e*^5^ = 6453.20175 Ω, where *V*_H_ is the Hall voltage, *I* is the current through the device, *R*_K_ is the von Klitzing constant, *h* is the Planck constant, and *e* is the elementary charge.

### 4.1 Measurements

Three different measurements were done to characterize the samples:

#### 1. Plateau Quality

The minima in *V_x_* (measured between pairs of contacts on the same side of the Hall bar) and the values of *V*_H_ (measured between pairs of contacts on opposite sides of the Hall bar) were measured with 25 μA flowing through the source and drain contacts (see Fig. 1b). The measurement system used in this work is similar to that described in Ref. [12]: an electronic current source drove a current through the Hall device connected in series with a room-temperature 10 kΩ reference resistor (Serial Number GR99). A high-quality standard cell scanner was used to both reverse the current direction and connect a high-stability 8.5 digit digital voltmeter (DVM) alternately between the reference resistor and the Hall resistor. The measurement system was entirely under computer control. Probe voltage measurements at a given magnetic field were made with current flowing alternately in opposite directions (i.e., when the drain was positive relative to source, the current was + 25 μA and when the drain was negative relative to the source, the current was − 25 μA). The voltages measured with current flowing in each direction were averaged to eliminate the contribution of thermal voltages to the measured voltages [12]. While this measurement system is capable of achieving quite low uncertainties, as described in Ref. [12], several modifications were made to the system to permit more rapid testing of samples. These modifications included the use of the electronic current source which had lower leakage resistances between its terminals and earth and higher noise than the mercury battery-powered current source used in that work, and the use of shorter measurement times. The resolution of the measurement system was about 0.05 μV for *V_x_* measurements, and the standard uncertainty due to random effects was about 0.05 μV (which corresponds to 2 mΩ at the 25 μA measurement current). For *V*_H_ measurements, the system had a resolution of about 0.1 μV (about 0.6 × 10^−6^ times the Hall resistance *R*_K_/4), and a standard uncertainty due to random effects of about 0.2 μV (about 1.2 × 10^−6^ times the Hall resistance). The combined standard uncertainty, including systematic effects, was of the order of 1 μV (about 6 × 10^−6^ times the Hall resistance).

#### 2. Contact Resistances

With the magnetic flux density set to a value at the middle of the *i* = 4 plateau (between 4.9 T and 5.3 T), the contact resistance of each contact was measured using a 3-terminal technique similar to that described in Ref. [4]. A programmable current source passed current between the contact of interest (denoted “A”) and a second contact (denoted “B,” usually the source or drain), and the potential was measured between the contact of interest and a third contact (denoted “C”) that did not carry current and was nominally at the same potential (i.e., on the same side of the Hall bar) as the contact of interest. The contact resistance was determined by measuring the voltage *V*_AC_ with the current set at one value (*I*_AB_), then increasing the current by an increment Δ*I*_AB_, measuring the voltage *V*_AC_(*I*_AB_ + Δ*I*_AB_), and dividing the *difference* in voltages by the current increment. In other words, the “dynamic” contact resistance was calculated using the formula:

$$R_{\text{AB,AC}}(I)=\frac{1}{\left[dI_{\text{AB}}/dV_{\text{AC}}\right]}\approx \frac{V_{\text{AC}}(I_{\text{AB}}+\Delta I_{\text{AB}})-V_{\text{AC}}(I_{\text{AB}})}{\Delta I_{\text{AB}}}$$ (1)

This definition of contact resistance was chosen for several reasons. Firstly, 1/*R*_AB,AC_ is the slope of the *I*(*V*) curve, which gives information about the nature of the potential barrier between the metal and the semiconductor, and is commonly used to assess deviations of the contact’s behavior from the ideal [31].

Secondly, the measured voltages are usually very small, particularly when small currents are passed through the device, and can be less than thermal voltages between the voltage probes. If the common definition of “static” contact resistance, i.e., *V*_AC_(*I*_AB_)*/I*_AB_, is used, nonnegligible thermal voltages can give rise to significant errors in the contact resistance. Because two voltages are subtracted to determine the “dynamic” contact resistance defined in Eq. (1) above, the thermal voltages cancel out, as long as they do not drift significantly between voltage measurements. Since the voltage measurements are made sequentially over a short period of time (less than 1 min) the thermal voltages will very likely not drift significantly, and their effect will cancel. It is for these two reasons that the “dynamic” contact resistance was used as a measure of contact resistance in this work.

In order to compare the “static” and “dynamic” contact resistances, however, the “static” contact resistances were also determined. The effect of the thermal voltages on the “static” contact resistance can be eliminated by measuring the voltage with the current set to zero (which voltage should equal the thermal voltage). The time required to measure the contact resistance over the range of current between − 100 μA and + 100 μA is significantly increased if one has to measure the thermal voltages every time a measurement is made; and if a single measurement of the thermal voltage is made, for example at the start of the experiment, one has to assume that it does not drift significantly during the time required to measure the contact resistance over the entire current range.

In this work, the thermal voltage was measured at the start of the experiment, and subtracted from all subsequent voltage measurements. The “static” contact resistance was calculated, in addition to the “dynamic” contact resistance defined in Eq. (1). In most cases, the “static” and “dynamic” contact resistances were quite similar in value and in their dependence on current, but the “static” contact resistances were considerably less accurate at small currents, due to drifts in the thermal voltage. The “dynamic” contact resistances, however, are much more sensitive to changes in the slope of the *I*(*V*) curve, such as occur when the contact “breaks down” when high currents are passed through it. In this case the changes in the “dynamic” contact resistance were always greater than those in the “static” contact resistance.

In all experiments, the contact resistances were measured over a range of current from − 100 μA to + 100 μA. In the earliest experiments, the current was initially set to − 100 μA, and increased in increments of between 0.5 μA and 10 μA until the maximum current was reached. It was found that the *R*_AB,AC_(*I*) curves generated using this measurement method tended to be asymmetrical: specifically, the absolute value of the critical current at which the resistance increased sharply tended to be slightly smaller for negative currents than for positive ones. This can be see in Fig. 6d, for example, where the negative critical current is − 25 μA, while the positive critical current is + 30 μA. In more recent experiments the measurement procedure was changed: the current was initially set to 0 μA, and the contact resistances determined as the current was increased to + 100 μA; it was then reset to 0 μA, and the measurements repeated as the current was decreased to − 100 μA. With this measurement procedure, in most cases the current dependence of the contact resistance was much more symmetrical, and the critical currents were the same for positive and negative current directions (see Fig. 6a).

It should be noted that the resistances measured using this technique included both the resistance of the contact and the resistance of all the wires connecting it to the connector at the top of the probe and the measurement system. This combined wire resistance was typically between 1.2 Ω and 1.9 Ω. Contact resistances were measured with a standard uncertainty of 0.3 Ω.

#### 3. Critical Current

Also, with the magnetic flux density set to a value at the middle of the *i* = 4 plateau, current was passed through the source and drain contacts at the ends of the Hall bar, and the voltage between a pair of contacts on the same side of the Hall bar (*V_x_*) was measured as a function of current. The current at which *V_x_* increases sharply, called the critical breakdown current, was measured.

### 4.2 Quality Criteria

The quality of the samples was rated according to the following factors:

#### 1. Plateau width

**Fig. 4:**
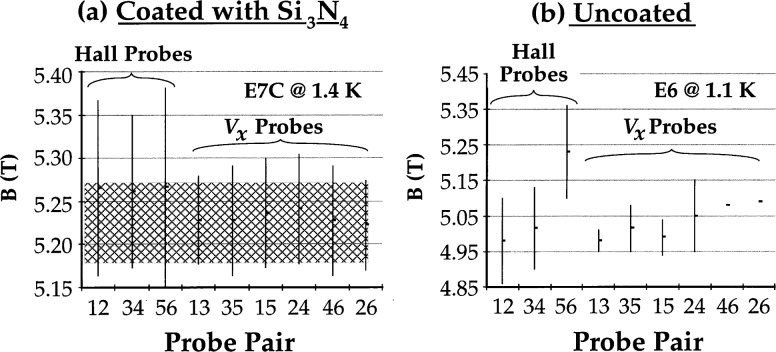
Regions of magnetic flux density over which the different Hall voltages exhibit plateaus and the *V_x_* voltages measured between probes on the same side of the Hall bar exhibit minima. The pairs of numbers along the horizontal axis indicate the two probes (cf. Fig. 1b) between which the voltages were measured: for example, “15” represents the voltage measured between probes 1 and 5. (a) Data for sample E7C, a sample covered with a protective silicon nitride coating. The grey rectangle indicates the “overlap region.” (b) Data for E6, a sample without a protective silicon nitride coating.

The minima in *V_x_* and plateaus in *V*_H_ should extend over as large a range of magnetic flux density as possible, and the plateaus observed in Hall voltages and minima in *V_x_* voltages measured between different probe pairs should occur, to as great an extent as possible, over the same range in magnetic flux density (this range of magnetic flux density is referred to as “plateau overlap” or simply “overlap” in this paper; see Fig. 4).

#### 2. Plateau values

**Fig. 5:**
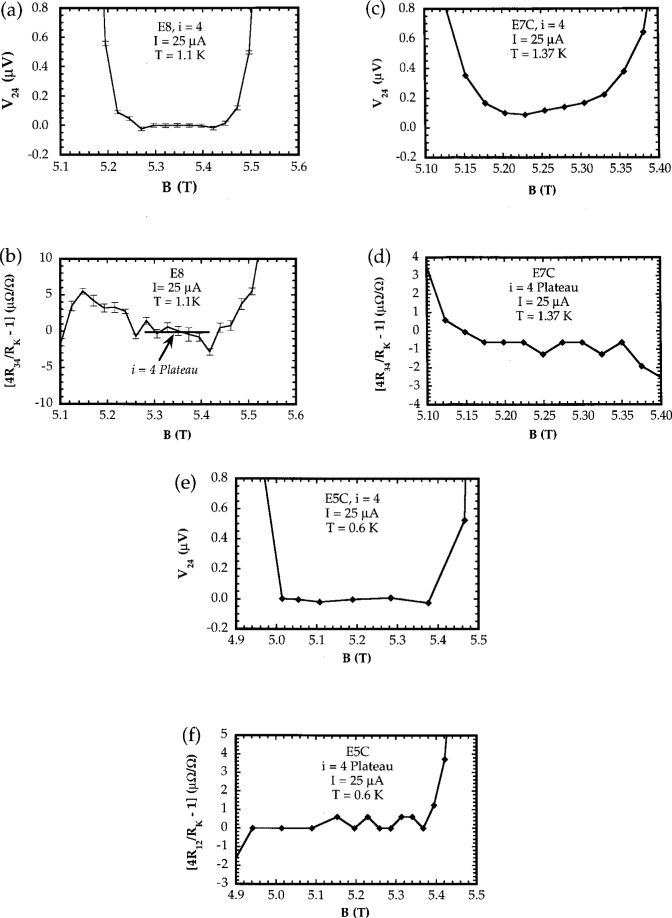
Graphs of *V_x_* and the difference between the Hall voltage and its ideal value (*IR*_K_/4) as a function of magnetic flux density for the *i* = 4 QHE condition. A current of 25 μA was flowing through the source and drain contacts. (a, b) Data taken from uncoated sample E8 at 1.1 K. (c, d) Data taken from coated sample E7C at 1.37 K. Graphs (a, b) and (c, d) are similar to those obtained from the other unpassivated and passivated devices, respectively. (e, f) Data taken from coated sample E5C at 0.6 K. Note that the approximately 0.6 μΩ/Ω difference between the measured Hall voltage and its ideal value at the center of the plateau in (d) and the variations in the Hall resistance in (d) and (f) are less than the uncertainty of measurement and at or near the limit of resolution of the measurement system, and cannot be considered to be significant on the basis of these measurements.

On the plateaus, the values of *V_x_* measured between probes on the same side of the sample should vanish to within the uncertainty of the measurement system. Values of *R*_H_, defined as *V*_H_/*I*, where *V*_H_ is the Hall voltage and *I* is the current flowing through the current contacts of the sample, should equal the ideal value of *h*/4*e*^6^ = 6453.20175 Ω. Figure 5 shows typical plots of the dependence of *V_x_* and deviation between *R*_H_ and its ideal value on magnetic flux density under the *i* = 4 QHE conditions for unpassivated (Fig. 5a, 5b) and passivated (Fig. 5c, 5d) samples at temperatures near 1 K. Figures 5e and 5f show corresponding plots for one of the samples coated with silicon nitride measured at 0.6 K.

#### 3. Contact resistances

**Fig. 6:**
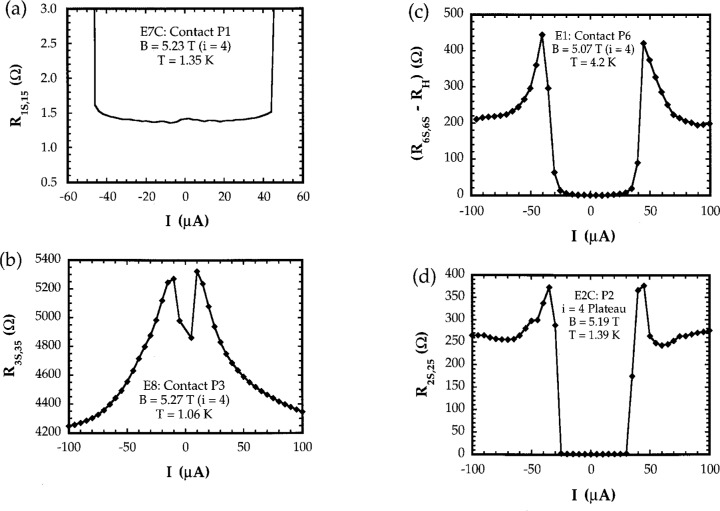
Comparison of current dependence of potential probe “dynamic” contact resistances (including probe wire resistance) on coated and uncoated samples measured at the *i* = 4 QHE condition. (a) Probe P1 on sample E7C (coated with silicon nitride); (b) Probe P3 on E8 (uncoated); (c) Two-terminal “dynamic” resistance of probe P6 of sample E1 (uncoated) at 4.2 K with the Hall resistance *R*_H_ = *R*_K_/4 subtracted out; this resistance includes the contact resistance of the source, which was negligible over the current range shown in this graph; (d) Probe P2 of sample E2C (coated with silicon nitride).

Ideally, all contacts should have contact resistances less than a few milliohms under QHE conditions, otherwise the measured values of *R*_H_ may be noisy and may deviate slightly from the ideal value of *h*/4*e*^2^. In practice, it has been found that in some cases, samples can still be used as resistance standards even when the resistances of some of the potential probes differ from zero by as much as a few hundred ohms [1]. The more contacts that have low resistance, however, the easier the sample is to use, and the greater its reliability. Graphs showing typical current dependences of contact resistances of good and bad potential probe contacts are shown in Fig. 6.

#### 4. Critical breakdown currents

**Fig. 7:**
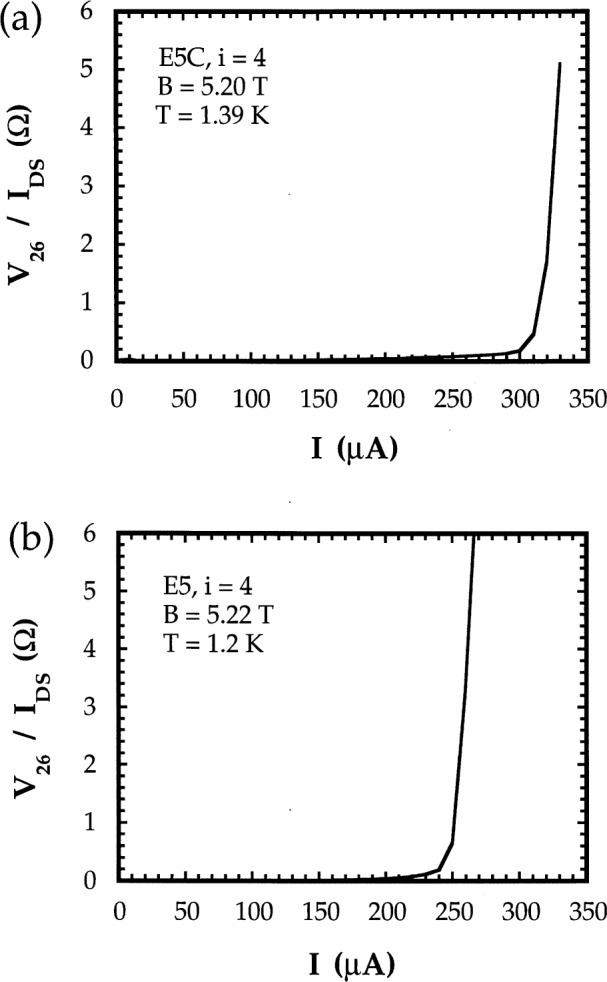
Comparison of critical breakdown currents in (a) coated and (b) uncoated samples measured under the *i* = 4 QHE condition. The graphs show the dependence of *V*_26_/*I* on current.

At currents above the critical current, *V_x_* values depart rapidly from 0 (see Fig. 7) and at high enough currents, *R*_H_ deviates from the ideal value of *h*/4*e*^2^, making the device unusable as a resistance standard [13]. The higher the current that can be used during measurements, the higher the signal-to-noise ratio, and the shorter the averaging time required to obtain a given uncertainty in the measurement. Thus, in order to be most useful as a resistance standard, a sample should have as high a critical current as possible.

In the next section, Sec. 4.1, the results of the tests on the two samples with passivating silicon nitride coatings, denoted by the serial numbers E5C and E7C are summarized. Section 4.2 summarizes the results of the tests on the four samples without passivating nitride coatings, denoted E5, E6, E7, and E8.

### 4.3 Passivated Samples

The characteristics of the passivated samples measured under QHE conditions at 1.4 K are summarized in Table 1. All contact resistances were vanishingly small (see Fig. 6a and 6d) and the plateaus were reasonably well centered about a common value (Fig. 4a). As can be seen, however, even at about 1.4 K, the minima in *V_x_* do not vanish (Fig. 5c) and the deviations of the Hall voltages from their ideal value (Fig. 5d) are not negligible, as they must be if the sample is to be used as a resistance standard [3]. The plateau values reported in Table 1 are consistent with measurements on similar samples reported by Piquemal and coworkers [1].

**Fig. 8:**
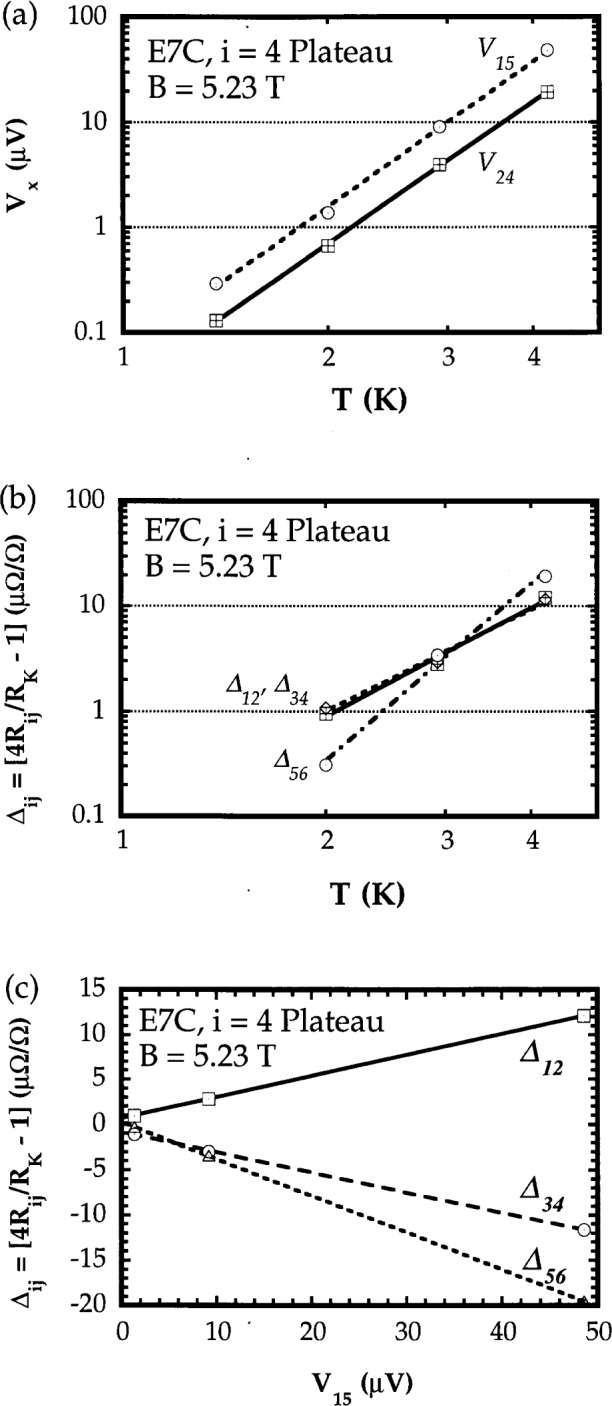
Temperature dependence of (a) the minimum in *V_x_* and (b) the absolute value of the difference between the Hall resistance at the center of the *i* = 4 plateau and its nominal value of *R*_K_/4 =*h*/4*e*^2^ measured between different probe pairs on sample E7C between 1.4 and 4.2 K. (c) The difference between the Hall resistances measured between different probe pairs and the ideal value of *R*_K_/4 is a linear function of the value of *V_x_*. In these figures, the symbols *V_ij_*, where *i* and *j* are integers, refer to the voltages measured between probes *i* and *j* (see Fig. 1b for probe numbering).

The minimum value of *V_x_* and the value of *R*_H_ at the magnetic field at which *V_x_* was minimum both exhibited a strong temperature dependence. This is illustrated in Fig. 8, which shows a graph of the values of the minima in *V_x_* and the deviations of the Hall voltages measured between different probe pairs on the Hall bar from the ideal value of *IR*_K_/4 (where *I* is the current through the device and *R*_K_ = 25812.807 Ω is the von Klitzing constant) determined at the magnetic flux density at which *V_x_* was minimum, as a function of temperature between 1.4 K and 4.2 K. While Fig. 8a shows only the voltages measured between probes 1 and 5 and 2 and 4 (see Fig. 1b for probe numbering), the voltage between probes 2 and 6 was identical to that between 1 and 5, and the voltages between the other potential probes (1 and 3, 3 and 5, and 4 and 6) were indistinguishable from the voltage between probes 2 and 4. Therefore these data were omitted from the graph. The difference between the *V*_15_ data and the *V*_24_ data is nearly exactly a factor of 2, reflecting the fact that probes 1 and 5 are separated by 1 mm, whereas probes 2 and 4 are separated by only 0.5 mm, half the distance. The deviation of each Hall resistance from the ideal value of *R*_K_/4 at a given temperature is a linear function of the minimum value of *V_x_* measured at that temperature, as shown in Fig. 8c, in agreement with Ref. [14].

Both samples were subsequently tested at 0.3 K, and the plateaus were found to be much broader, the *V_x_* values vanished, the Hall plateaus had values equal to their ideal value, and the samples were usable as resistance standards at the lower temperature. Figures 5c and 5d, and 5e and 5f show traces of the minimum in *V_x_* and the Hall plateau on samples E7C and E5C respectively, as functions of magnetic field at 1.4 K and 0.6 K, respectively. The minimum in *V_x_* is clearly nonzero at 1.4 K, but vanishes at 0.6 K and below, and the plateau in *V*_H_ and minimum in *V_x_* extend over a greater range of magnetic field at 0.6 K than at 1.4 K. While the graphs in Fig. 5 only show data taken from selected voltage probes, the data from the other voltage probes on these samples were practically identical.

### 4.4 Unpassivated Samples

The results of the tests of the unpassivated samples are summarized in Table 2. The ranges of magnetic field over which the plateaus were observed is similar in both cases, but, as shown quite graphically in Fig. 4, the range of magnetic field over which all plateaus in *V*_H_ and minima in *V_x_* on a given sample coincided (referred to as the “overlap region”) was rather less for the uncoated samples than for the coated ones. It should be noted that the lack of “overlap” exhibited by sample E6 as shown in Fig. 4, was the worst of any of the samples tested. The plateaus measured on the other uncoated samples generally overlapped to a much larger degree: on E7, the “overlap region” for all plateaus save a single Hall plateau was about 0.1 T, quite comparable to the coated samples^5^; and the “overlap region” of 5 of the plateaus on E8 was also about 0.1 T, with 2 Hall plateaus and 2 *V_x_* minima occurring over a range of magnetic flux density that was approximately 0.1 T lower, resulting in the low combined overlap of 0.05 T reported in the table.

Also, in contrast to the passivated samples tested at 1.4 K, the minima in *V_x_* measured on the unpassivated samples all vanish to within the uncertainty of measurement, except in the two cases noted in the table. This is illustrated by a comparison of Figs. 5a and 5c. Figure 5a shows a representative graph of the magnetic field dependence of a *V_x_* measured on the uncoated sample E8, and Fig. 5c shows the same graph, but measured on sample E7C, coated with silicon nitride. One can clearly see that the *V_x_* minimum is quite broad and vanishes to within the measurement uncertainty of about 50 nV on the uncoated sample, while the *V_x_* measured on the coated sample does not vanish at any magnetic field. These traces are typical of the *V_x_* vs B behavior observed on the other coated and uncoated samples. It is interesting to note that despite the fact that the contact resistances were rather high on sample E8, the *V_x_* minima did vanish to within the measurement uncertainty.

The Hall resistances determined from Hall voltage measurements made between different probe pairs on each uncoated sample were also equal to their ideal values except in the case of two of the three Hall probe pairs on sample E8 (*V*_12_ and *V*_56_) and one of the three Hall probe pairs on E7 (*V*_12_). The Hall plateau measured between probes 3 and 4 on sample E8, shown in Fig. 5b, is representative of the good plateaus obtained on the uncoated samples. In contrast to the Hall plateaus measured on the coated samples (see Fig. 5d for a representative trace), those on the uncoated samples were somewhat narrower.

The critical current at which breakdown of the quantum Hall effect occurred in the unpassivated samples was lower than in the passivated samples. This is illustrated in Fig. 7, which shows representative traces of the voltage measured on passivated (Fig. 7a) and unpassivated (Fig. 7b) devices between potential probes 2 and 6 with the magnetic field set to the center of the *i* = 4 plateau as a function of the current flowing through the current contacts (source and drain) at the ends of the Hall device (the measured voltages have been divided by the current to give the resistance plotted in the figure). These traces were quite typical of those measured on all the other devices tested: the critical currents varied by ± 10 μA or less from sample to sample. It is interesting that while the critical currents of the coated samples measured in this work agreed with those reported in Ref. [1], the critical currents of the uncoated samples were less than those on the coated samples, rather than greater as reported in Ref. [1].

All but two of the contacts on the unpassivated samples were of worse quality than any of the contacts on the passivated samples. The poorer contact quality was manifested in either of two ways: in some cases, the contact resistances did vanish, but only for currents less than 10 μA (and in some cases even less than 5 μA); in other cases, the contact resistances did not vanish at all. Of the current contacts, only the drain on E5 had zero resistance up to 100 μA (the maximum current used in the test); the source and drain contacts on the other samples had minimum resistances from 6 Ω to several hundred ohms. Of the potential probe contacts on the unpassivated samples that exhibited zero contact resistance only two (probe P1 on E5 and P2 on E6) did so at currents approaching 40 μA; the others only exhibited zero contact resistance at currents below about 10 μA. This is in marked contrast to the contacts of the passivated samples, the source and drain contacts of which uniformly had zero contact resistance to currents above 100 μA, and the potential probes of which had contact resistances that uniformly vanished below currents of between 35 μA and 45 μA.

This is illustrated in Fig. 6, which shows representative graphs of the dependence of contact resistance on current for potential probe contacts on the coated and uncoated samples. Figure 6a illustrates the behavior typical of all the potential contacts on the passivated samples. The data in Fig. 6a were obtained as described in the second paragraph of Sec. 4.1, and include the resistance of the wires connecting the sample to the connector at the top of the cryostat and to the rest of the measurement system, which in this case was about 1.4 Ω. The sharp increase in contact resistance at currents above 45 μA shown in Fig. 6a is most probably due to breakdown of dissipationless current flow in the neck of the potential probe: the ratio of the critical channel current (cf. Fig. 7) to the device width (300 μA/400 μm) is approximately the same as the ratio of the current at which the probe contact resistance increases sharply to the width of the probe neck (40 μA/50 μm).

In contrast, the current dependence of the contact resistance of one of the worst contacts on sample E8 is illustrated in Fig. 6b, which shows the voltage measured between probes 3 and 5 divided by the current flowing between probes 3 and the source as a function of current, with the magnetic field set at the center of the *i* = 4 plateau. While this contact was the second worst (only one other contact on all the samples tested, P1 on sample E8, had higher contact resistance, and most of the other contacts had resistances an order of magnitude or more less than that shown in Fig. 6b), it does illustrate the general behavior of the current dependence of the poor contacts on the uncoated samples.

The current dependence of the contact resistance shown in Fig. 6b is quite significant, for it has exactly the same form that one would observe if very high currents were passed through a good contact. This is illustrated in Fig. 6c, which shows the current dependence of the contact resistance of a good contact on unpassivated sample E1. Sample E1 was mounted in 1990, shortly after the LEP samples were received, by attaching gold wires directly to the contacts on the heterostructure using small beads of indium in such a manner as to avoid applying any pressure to the heterostructure that might create electrically active defects. This sample was tested in December, 1990, and quite interestingly, the behavior of the contact resistances on this uncoated sample at that time was very similar to that of the contact resistances on the coated samples. Even at temperatures as high as 4.2 K, *all* contact resistances vanished to within the resolution of the measurement system: the source and drain contact resistances vanished at currents up to 100 μA (the highest current used in the test), while every potential probe contact resistance vanished at currents less than 20 μA to 30 μA. At higher currents, the contact resistance increased, but then decreased slightly, as shown in Fig. 6c. While the data in the figure were taken at 4.2 K, the same general dependence is observed at lower temperatures, except that the changes in slope of the curve tend to be much sharper (cf. Fig. 6d) and the range of current over which the contact resistances vanish is somewhat larger.

This behavior is observed in the contact resistances of the coated samples as well, and is independent of the method used to attach wires to the sample. Figure 6d shows the current dependence of the resistance of a typical contact on a coated LEP sample, identified as E2C, mounted and tested at about the same time as E1. Wires were attached to two of the contacts on this sample, P1 and P2, by carefully bonding the wires directly to the bonding pads; wires were attached to the rest of the contacts using small beads of indium, as was done with sample E1. The data shown in Fig. 6d were taken from one of the wire-bonded contacts and illustrate several important points. First, the current dependence of the resistance of the wire bonded contact shown in Fig. 6d was essentially the same as that of the contacts to which wires had been attached using indium beads, so this behavior is, as mentioned above, independent of the method used to attach wires to the sample and furthermore is not affected by the silicon nitride coating. Second, comparison of the contact resistances of E2C and E7C, shown in Figs. 6d and 6a, respectively, indicate that the contacts on the coated samples have changed little over the 6 years since the samples were received, quite in contrast to the situation with the uncoated samples (Figs. 6b and 6c). Lastly, it should be noted that the “critical current” below which the resistance of contact P2 on E2C (shown in Fig. 6d) vanished was only about 25 μA and that for contact P1 was about 5 μA, about 10 μA to 30 μA *lower* than the “critical current” for the other contacts on that sample, which had wires attached with indium beads. The “critical currents” for the contacts to which wires had been attached with indium beads were between 35 μA and 45 μA, which was the same as the “critical currents” of the contacts on E7C, to which wires had been bonded over the substrate. The lower “critical currents” of the contacts to which wires had been bonded directly over the heterostructure on E2C are a manifestation of damage to the contact and heterostructure created by the stresses induced when the wires were bonded [4].

Significantly, the general features of the current dependence shown in Figs 6c and 6d is exactly the same as those exhibited by the contact P3 on sample E8 (Fig. 6b), except that the resistance of that contact does not vanish at low currents and the resistances are all much higher than on the good contacts. Indeed, all of the contacts on all of the samples tested exhibit a common behavior: the resistances of the best contacts vanish over some range of current, then increase sharply at some “critical current,” and then decrease again for currents greater than this “critical current,” as shown in Figs. 6c and 6d. The “critical current” for the best contacts (cf. Fig. 6a) is over 40 μA, and decreases as the quality of the contact decreases. A limiting case is reached when the “critical current” is essentially 0 μA, in which case the contact resistance vs current curve is similar to that shown in Fig. 6b, except that the minimum in the contact resistance curve at *I* = 0 is 0. As the contact quality gets worse, the minimum in the contact resistance curve at *I* = 0 does not vanish and becomes larger the poorer the contact, as is shown in Fig. 6b. The second limiting case is reached when the contact quality is so poor that no minimum is exhibited at all: the contact resistance simply increases to a maximum at *I* = 0. Interestingly, while this second limiting case is usually realized in situations in which the magnitude of the contact resistance is very high (thousands of ohms), it can occasionally be realized in situations in which the actual value of the contact resistance is not terribly high: for example, in the case of the source contact on uncoated sample E5, the maximum resistance at low currents never exceeded 12 Ω, and the contact resistance decreased to a minimum of less than 5 Ω at 50 μA.

Contact resistances with current dependence like this (i.e., reaching a maximum at zero current) or like that shown in Fig 6b were not observed on E2C, E5C, E7C, or E1, but were observed on nearly all uncoated samples (E5 … E8) tested in 1996. Significantly, such behavior was also observed on the contacts of one coated sample, designated E6C, that was mounted and tested in 1992. Wires were attached to that sample by directly bonding 25 mm diameter gold wires to the Ti/Pt/Au bonding pads over the heterostructure. Tests on this sample were reported in Ref. [4], in the section entitled “Direct Wire Bonding.” In the case of this sample, the contact and the heterostructure beneath the bonded wire were clearly damaged by the high pressures created during wire bonding. Current dependent contact resistances like that shown in Fig. 6b, or which attained a maximum at zero current were attributed to mechanical damage to the contact or heterostructure during bonding, which created electrically active defects in the sensitive region between the contact and the heterostructure. Since the current dependence of the contacts on the uncoated samples E5…E8 tested in 1996 is similar, it seems reasonable to assume that this behavior is also due to damage to the contact, though in the case of E5… E8, the damage is not mechanical, (as bonding was performed over the substrate, and no force of any kind was applied to the heterostructure) but electrochemical in nature.

## 5. Causes of Degradation of Unpassivated Samples

It is apparent from the data presented in Sec. 4 that the quality of the ohmic contacts on the unpassivated samples is considerably worse than that of those on the passivated samples. In addition, the “overlap”—the range in magnetic flux density over which plateaus observed between different probe pairs on a single sample coincide—is somewhat smaller for the unpassivated samples than for the passivated ones. Since the range of magnetic flux density over which a plateau is observed is proportional to the local electron concentration in the 2-dimensional electron gas (2-DEG), the decreased overlap observed on the unpassivated samples indicates that the properties of the heterostructure that govern the concentration of electrons in the 2-DEG have been changed on the unpassivated samples. Section 5.1 discusses the possible causes of the differences in the quality of the ohmic contacts on the passivated and unpassivated samples. The origin of the increased inhomogeneity in electron density in the 2-DEG observed in the unpassivated samples is discussed in Sec. 5.2.

### 5.1. Contact Degradation

Since the passivated and unpassivated samples were presumably both prepared by LEP at the same time using the same procedure, it is likely that the quality of the contacts on the passivated and unpassivated devices were identical at the time of manufacture. This assumption is in agreement with the observation that the resistances of the contacts on the unpassivated sample (E1) tested in 1990 shortly after receipt of the samples were identical to those of the contacts on a passivated device (E2C) tested at the same time (Figs. 6c and 6d). Furthermore, because essentially the same procedure was used to mount both the passivated and unpassivated samples (i.e. E5C and E7C, and E5–E8), it is very unlikely that some step in the mounting procedure was responsible for the observed differences in their contact resistances. Specifically:

- Both types of samples were exposed to the same solvents, and if anything, the passivated samples were exposed to more ultrasonic agitation than the unpassivated ones, so it is unlikely that the cleaning procedure had any significant deleterious effect on the contacts.
- All bonding pads were deposited in the same evaporator under nearly identical conditions, so it is unlikely that the high resistances of the contacts on the unpassivated samples are due to an insulating film between the bonding pad and the alloyed contact;
- In all cases, wires were bonded to the bonding pads over the substrate, and *not* over the heterostructure, so it is most unlikely that there was any damage to the heterostructure from bonding or that it had any effect on the contact resistances;
- With the exception of E8, all samples were exposed to the 200 °C bonding temperature for about the same amount of time, so it is unlikely that exposure to a high temperature caused a chemical change in the contacts that resulted in higher resistance.^6^

Since both the passivated and unpassivated samples were mounted using essentially identical processes, and were stored under identical conditions, the difference in contact quality can only be due to some mechanism that acted differently on the two types of samples by virtue of some difference in their design. The most obvious difference between the passivated and unpassivated samples is that the former are covered entirely with a 165 nm thick coating of silicon nitride. The nitride covers not only the GaAs/AlGaAs heterostructure, but *also* covers the *edges* of the alloyed AuGe/Ni ohmic contacts; the rest of the alloyed contacts are protected by the fairly thick Ti/Pt/Au bonding pad. While the alloyed AuGe/Ni contacts on the unpassivated samples are also partially covered with the Ti/Pt/Au bonding pad (see Fig. 1c), the *edges* of the alloyed contacts are freely exposed to the atmosphere.

The quality of the alloyed AuGe/Ni contacts is critically dependent upon the condition of the edges of the contacts that abut the heterostructure. When the AuGe/Ni film is heated during formation of the contact, the alloy melts and dissolves the heterostructure beneath it. If the alloying time is too short, the alloy will only partially penetrate the AlGaAs donor layer. Since the electron concentration in this donor layer is negligibly small at the temperatures at which the quantum Hall effect is observed, the layer of AlGaAs between the metal and the GaAs channel in which the 2 DEG resides serves as an insulating barrier. The high tunneling resistance of this barrier will cause the contact resistances to be very large. Minimum contact resistances are obtained when the alloying time is long enough for the metal to reach the proximity of the interface between the AlGaAs donor layer and the GaAs channel in which the 2 DEG resides [15, 16]. The penetration of the metal to this interface, however, alters the potential well in which the electron gas is confined and reduces the density of electrons in the 2 DEG immediately below the contact. Lateral injection of electrons from the edge of the metal contact into the 2 DEG then contributes significantly to conduction [17] and any alteration of the structure of this region of the contact, due either to chemical change caused by corrosive agents or mechanical damage, can have a disproportionately large effect on the resistance of the contact.

The alloyed AuGe/Ni contacts are quite thin, being less than about 300 nm in thickness, and are chemically quite complex. The resistances of these contacts are extremely sensitive to changes in the chemical structure of the contact: exposure of the contacts on unpassivated samples to mildly caustic solutions such as photoresist remover for just 5 min was found to cause the contact resistances to increase to > 17 kΩ. Commercially available cleaning solutions containing about 1.5 % choline were found to greatly increase the contact resistances by as much as several megohms after about 10 minutes exposure. Photoresist developer also measurably increased the contact resistances, although not as drastically (by between zero and a few hundred ohms after between 1 min and 5 min at room temperature). Since the contacts contain alloys of Ni, Ge, As, and Ga [18] in the presence of Au, which is an electrochemically more noble metal, it seems likely that electrochemical corrosion of one or more constituents of the contact can occur, which in turn can change the chemical structure of the contact at the metal-semiconductor interface and increase its contact resistance. The contacts on the passivated samples take somewhat longer to degrade than do the contacts on unpassivated samples in these solutions, indicating that the silicon nitride coating does reduce the access of corrosive chemicals to the sensitive edge region of the contact abutting the heterostructure.

While none of the samples tested in this study (viz. E5C, E7C, and E5… E8) were exposed to such corrosive solutions, they were stored in an unregulated laboratory environment, in which the temperature varied over a wide range, between 18 °C and 30 °C, and the relative humidity varied between 10 % and 70 %. Under these conditions, at least several monolayers of water molecules are adsorbed by the surface of the sample. In the presence of even low concentrations (less than 10^−4^ %) of common atmospheric contaminants such as chlorine, corrosion has been observed to occur [5]. The exposed alloyed contacts on the unpassivated samples permitted the corrosive agents ready access to the sensitive regions of the contacts. Furthermore, in virtue of the small volume of the sensitive region of the contacts, even small amounts of corrosion could have a disproportionately large effect on the chemistry and resistance of the contacts. The silicon nitride coating on the passivated samples appears to have effectively prevented the corrosive agents from gaining access to the alloyed contacts, and prevented the degradation of the contacts on those samples.

### 5.2. Heterostructure Degradation

While the plateaus exhibited by the passivated and unpassivated samples occurred over essentially the same range of magnetic field (between 5.0 T and 5.4 T), the “overlap region” of magnetic field over which voltages measured between every pair of potential probes on a given sample exhibited plateaus or minima was somewhat smaller for the unpassivated samples than for the passivated ones, as is illustrated graphically in Fig. 4. While the data in Fig. 4 represent an extreme case, it does seem that the “overlap region” for the unpassivated samples is somewhat smaller than for the passivated ones, and hence the electron concentration in the 2-DEG is somewhat more nonuniform in the unpassivated samples than in the passivated ones.

This nonuniformity in electron concentration in the 2-DEG can be caused by several factors, including overly rapid cooling of the sample from room temperature to the cryogenic temperatures at which the QHE is observed, and nonuniform oxidation of the top layers of the heterostructure, which in the case of the unpassivated samples was freely exposed to the atmosphere. While the effects of rapid cooling cannot be neglected, it is unlikely that they alone are responsible for the difference in “overlap regions” for the unpassivated samples, for both the passivated and unpassivated samples were cooled in the same manner in the same cryogenic system. Furthermore, all samples were cooled as slowly as possible, generally leaving them overnight in a tube filled with helium gas in the cyrostat and cooled by conduction from a reservoir of liquid nitrogen before cooling them to liquid helium temperatures.

Since it is presumed that the passivated and unpassivated samples were manufactured in a similar manner, it is assumed that the devices had similar uniformity in electron density at the time they were made. Unfortunately, the tests of the samples mounted in 1990 (E1 and E2C) were performed at higher temperatures and with a less accurate measurement system than the subsequent samples, and were not tested as completely (not all probe potentials were measured). The data that were taken indicate that E1 had an “overlap region” of between 0.02 T and 0.05 T at 4.2 K, which is quite remarkably large, considering that the widths of plateaus in the Hall voltage and minima in *V_x_* decrease markedly with increasing temperature. This would seem to indicate that the uniformity of the electron density in the coated and uncoated samples was very similar at the time the samples were made, and the difference in “overlap regions” for the passivated and unpassivated samples measured in this study (in 1996) is due to some effect that has operated more vigorously on the uncoated samples to decrease the homogeneity of their electron concentrations.

The most likely such effect would be oxidation of the heterostructure, for this would occur more readily on the uncoated than on the coated samples. It is well known that GaAs surfaces exposed to the atmosphere will oxidize readily [19], with the formation of both gallium and arsenic oxides. The arsenic oxides, however, are not thermodynamically stable in the presence of GaAs, and over time react with the GaAs to form more gallium oxide and free arsenic at the oxide-GaAs interface [20]. The free arsenic can generate arsenic anti-site defects [21] that pin the Fermi level near the middle of the bandgap, depleting the electrons in the cap layer, particularly at low temperatures [22]. As a consequence, no current flows through the cap layer in parallel with the 2 DEG in the unpassivated samples, and the *V_x_* voltages measured between pairs of contacts on the same side of the Hall bar vanish. Furthermore, defects or impurities in the heterostructure can cause different localized regions of the heterostructure to oxidize at different rates, resulting in a spatially nonuniform density of traps that can deplete the donor layer as well as the cap layer in the vicinity of the defect. This can cause a nonuniform electron concentration in the 2 DEG in the unpassivated samples which would cause plateaus measured between different probe pairs to occur over different ranges in magnetic field, resulting in the observed decreased overlap shown in Fig. 4. The somewhat larger plateau overlap on the passivated samples than on the unpassivated samples indicates that the silicon nitride layer does prevent this nonuniform oxidation of the heterostructure.

## 6. Methods of Protecting the Contacts

These observations indicate that the degradation of alloyed AuGe/Ni contacts and the heterostructure can be prevented by protecting them from exposure to the atmosphere by covering them with a layer of a material that is impervious to the agents responsible for corrosion. There are two ways in which this can be done: the contacts alone can be covered with a protective film, leaving the rest of the heterostructure exposed as discussed in Sec. 6.1, or, as, discussed in Sec. 6.2, the contacts and the heterostructure can be coated with a thin, chemically impervious, insulating layer, like the silicon nitride used in the devices tested in this study.

### 6.1 Very Thick Bonding Pads

The degradation of the contacts observed in the unpassivated samples, if due to corrosion, can be prevented by coating the alloyed contacts alone with a film impervious to corrosive agents. English and Turner [5] found that corrosion of Au/Cr films on integrated circuits exposed to humid atmospheres containing 10^−4^ % of chlorine could be prevented by covering the metal films with a layer of gold greater than about 3 μm in thickness. Such a thick layer did not have any pin-holes, and adequately prevented corrosive agents from gaining access to the gold-chromium interface, where corrosion normally occurred.

If the mechanism responsible for the deterioration in the contacts on the unpassivated samples proposed here is correct, depositing a layer of gold greater than about 3 μm in thickness completely covering each alloyed contact, including its edges, would prevent corrosive agents from reaching the sensitive regions of the contact and hence would prevent their degradation. Deposition of such a thick layer of gold in such a manner that it completely covered the alloyed contacts and extended over the semi-insulating substrate would have several advantages: not only would it be easy to do, but the gold layer would absorb much of the stress generated when wires are bonded to the pad, minimizing the damage to the heterostructure and making it easier to bond wires to the devices. While this scheme should prevent the alloyed contacts from degrading, it will not prevent oxidation and associated degradation of the exposed heterostructure.

### 6.2 Silicon Nitride Passivation

The contacts and the heterostructure can both be protected from corrosion by covering the entire sample, save for parts of the bonding pads, with an insulating film. Of the possible materials that can be used, silicon nitride, deposited using a low-temperature chemical vapor deposition process, has been established as the best [23, 24]. Such a silicon nitride coating was used to coat the passivated samples used in this study. While the silicon nitride coating did preserve the contacts, however, these samples showed a residual resistance under QHE conditions that gave rise to nonzero *V_x_* values at 1.4 K (cf. Table 1) which make it difficult to use these samples as resistance standards at this temperature [3]. In order for this passivation technique to be useful, it must protect the contacts without introducing a conducting path in parallel with the 2 DEG, so the causes of this parallel conduction must be determined.

It has been supposed [1] that this nonzero *V_x_* value was due to parallel conduction through the protective nitride coating. Bulk conduction through the nitride layer, however, does not seem likely, for the bulk resistivity of silicon nitride is of the order of 10^14^ Ω cm [25]. The thickness of the silicon nitride coating on the samples used in this study was 165 nm. The resistance of a 0.5 mm wide by 1 mm long section of this nitride coating (the approximate dimensions of the nitride between the potential pads—see Fig. 1b) would therefore be about 10^19^ Ω, which is 15 orders of magnitude higher than the resistance of the device under the conditions required to observe the *i* = 4 quantum Hall plateau. Such a high resistance would not affect the electrical properties of the device, even when measured with the most sensitive measurement systems available today.

It also seems unlikely that conduction along the top surface of the nitride would be responsible for the observed parallel conduction: the surface conductance per square of SiO_2_ films even at room temperature is of the order of 10^−16^ S at a relative humidity of 30 % [26], a value that again, would not affect the measurements. Since one would expect the surface conductivity of silicon nitride to be less than that of SiO_2_, and since the surface conductivity decreases quite markedly as the temperature and relative humidity decrease, the surface conductivity of the silicon nitride should be negligibly small under the conditions used to observe the QHE.

Another possibility is that interface states between the GaAs cap layer and the silicon nitride coating give rise to a measurable conductivity. This possibility is hard to evaluate, for the exact method used to deposit the nitride is not known. If the nitride were deposited directly on an untreated GaAs surface, surface defects would generally give rise to energy levels quite deep within the bandgap [27] which would not be expected to contribute to any significant conduction, particularly under QHE conditions. These defects would prevent conduction at the silicon nitride-GaAs interface, but might not be sufficiently numerous to fully deplete the cap layer, possibly permitting a small current to flow through the cap layer.

If, however, the nitride were deposited in a hydrogen-ammonia plasma, native arsenic oxides would be removed, and gallium oxides converted to GaN, a wide bandgap semiconductor [28]. The surface defects that in unpassivated samples deplete the carriers in the cap layer would in this case not be present. In consequence, the cap layer, which has a fairly high donor density (≈ 4×10^17^/cm^3^) would remain undepleted and could possibly conduct current, even under QHE conditions, probably through a donor atom to donor atom “hopping” conduction mechanism. The current conducted through the cap layer would flow in parallel with the 2 DEG, and would give rise to the observed small nonzero *V_x_* voltages measured between pairs of contacts on the same side of the Hall bar (cf. Table 1). While the magnitude of the current is difficult to calculate, it would be expected to decrease rapidly with temperature, as observed (see Fig. 8).

If the parallel conduction is indeed due to current flow in the cap layer, one would have to etch away the cap layer after alloying the contacts and before depositing the passivating nitride coating in order to eliminate the parallel conduction (as was done by Bühlmann, Ref. [16]). Alternatively, a heterostructure with a lower donor density in the cap layer, or possibly even an undoped cap layer could be used to prepare the devices. An undoped cap layer would protect the AlGaAs from oxidation but should not conduct current in parallel with the 2 DEG under QHE conditions, even when covered with a passivating silicon nitride layer.

Both of these proposed methods should result in samples without significant *V_x_* values at 1.4 K, and which do not degrade with time. While coating the devices with a nitride layer requires considerably more complicated processing than simply depositing large, thick bonding pads as discussed in Sec. 6.1, properly passivated devices will prove to be more reliable, for both the heterostructure and the contacts will be protected by the nitride, and hence neither will be subject to corrosion.

## 7. Conclusion

Quantized Hall resistance devices made from GaAs/AlGaAs heterostructures with alloyed AuGe/Ni contacts, both with and without protective silicon nitride coatings have been mounted and tested under quantum Hall effect conditions. It was found that after storage in an unregulated laboratory environment for 6 years, the resistances of the contacts on the samples lacking the protective silicon nitride coating increased quite noticeably, while those of the contacts on the samples with the passivating silicon nitride coating had not. In addition, the electron density in the 2-DEG in the unpassivated devices was observed to be more nonuniform than that in the passivated devices. It was concluded that AuGe/Ni alloyed contacts exposed to air for long periods of time will experience electrochemical corrosion that will alter their chemical composition and increase their resistance. Exposure to air of the unprotected heterostructure on the unpassivated samples results in nonuniform oxidation of the heterostructure, giving rise to increased inhomogeneity in the electron concentration in the 2-DEG. This in turn causes a smaller overlap of the ranges of magnetic flux density over which the plateaus in the Hall voltages and minima in *V_x_* voltages measured between different probe pairs on a given device are observed.

While the samples with the passivating coating used in this study did not show any visible effects of degradation, they did exhibit the effects of a conducting path in parallel with the 2-DEG, probably due to conduction through the top cap layer at temperatures above 1 K which resulted in nonzero voltages measured between probes on the same side of the Hall bar, a condition which makes it difficult to use the samples as resistance standards. This parallel conduction was not observed in samples without the silicon nitride coating because the top surface of the heterostructure was exposed to the atmosphere and oxidized, resulting in the formation of surface defects that depleted the conducting electrons in the cap layer.

In view of the observations and these conclusions, two methods are proposed for preventing degradation of quantized Hall resistors with alloyed AuGe/Ni contacts. Gold films greater than 3 μm in thickness can be deposited over the alloyed contacts. Sufficiently thick films will not have pinholes, and if they completely cover the alloyed contact including its edges, they should adequately prevent corrosive agents from getting to the sensitive alloyed contact and degrading it. This technique is by far the simplest and easiest to implement, but will not prevent oxidation-induced degradation of the heterostructure. Degradation of the devices can also be prevented by coating them with a passivating silicon nitride coating. Parallel conduction in these passivated devices can very likely be eliminated by etching away the cap layer prior to depositing the silicon nitride, or by making the devices on a heterostructure with an undoped cap layer. While coating the devices with nitride is a technically somewhat more challenging task, it is certainly not impossible, and should result in more reliable devices, for both the alloyed contacts and the heterostructure itself will be protected from corrosion and oxidation by the nitride coating.

In summary, we can conclude:

1. Bonding pads at least 3 mm thick (and preferably thicker) must be deposited over both the alloyed AuGe/Ni contact AND the semi-insulating substrate. Wires must be bonded to the pad over the substrate to prevent the generation of electrically active defects in the heterostructure which increase the contact resistances [4].
2. The resistance of AuGe/Ni contacts on QHR devices that are NOT protected from the atmosphere with an impervious coating will increase with time.
3. The uniformity of the electron concentration within QHR devices that are NOT protected with an impervious Si_3_N_4_ coating will decrease with time, resulting in a variation in the range of magnetic flux density over which quantum Hall effect (QHE) plateaus measured between different probe pairs are observed.
4. Covering both the contacts and the heterostructure with an insulating film, such as silicon nitride, that is impervious to humidity and other atmospheric contaminants will prevent both from degrading with time.

## Figures and Tables

**Fig. 3(a):**
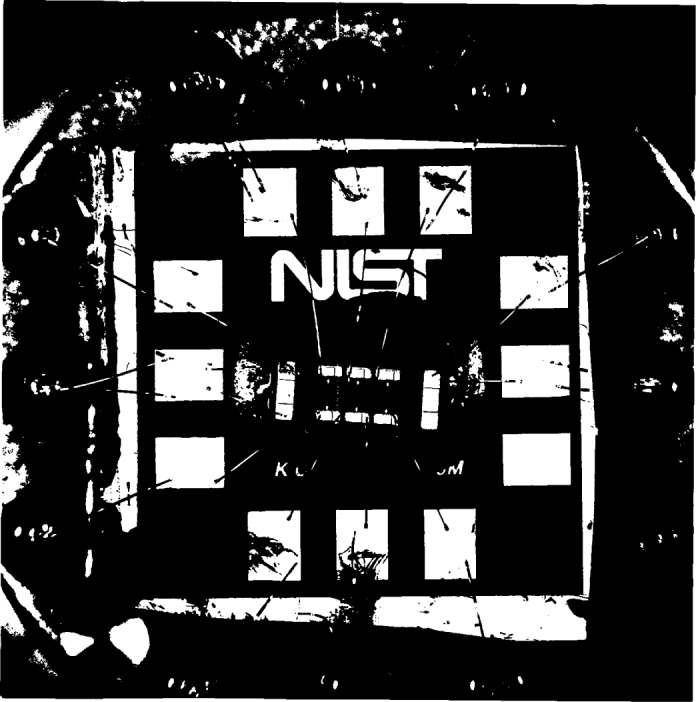
Photograph of sample E8 mounted on a glass carrier plate in a TO-8 header using the procedure described in Sec. 3.

**Fig. 3(b):**
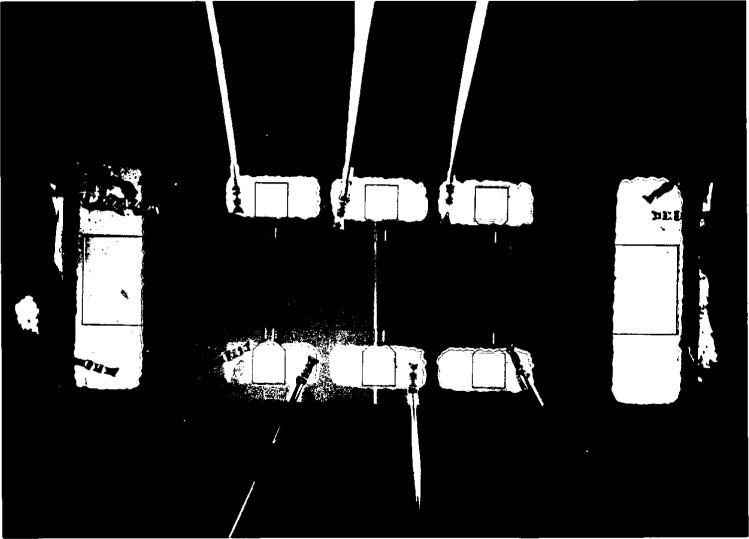
Enlarged view of sample E8 showing the enlarged bonding pads and the wires bonded to them.

**Table 1:** Summary of properties of passivated samples at 1.4 K

Sample	Plateau centers, width and overlap^a^	*R_x_* = *V_x_*/*I*	Hall plateau value (4*R*_H_/*R*_K_−1)	Breakdown current	Contact resistances
E5C	Center: 5.12 T; Widths: 0.05 T to 0.3 T Overlap^b^: 0.02 T to 0.12 T	[(5 to 6) ± 3] mΩ @ 1.4 K; 0 mΩ @ 0.5 K	[(−0.1 to −0.7) ± 0.8] × 10^−9^ @ 0.5 K^c^	300 μA	All^d^ 0 for *I* < 35 μA to 45 μA
E7C	Center: 5.23 T Widths: 0.1 T to 0.25 T Overlap: ≈ 0.1 T	[(3 to 15) ± 3] mΩ	[(0.3 to 1.5) ± 1.5] × 10^−6^	320 μA	All^d^ 0 for *I* < 35 μA to 45 μA

a The plateaus in the voltages measured between different probe pairs on the same sample were sometimes centered at slightly different values of the magnetic field, and often different plateaus had different widths. There was, however, a range of magnetic field over which the voltages measured between all probe pairs exhibited plateaus. This range is called the “overlap.”

b The sample was cooled and tested twice: the first time the overlap was only 0.2 T; the second time, the sample was cooled more slowly, and the overlap was 0.12 T.

c Accurate measurements of the Hall voltages on sample E5C at 1.4 K were not made. The sample was tested at 0.5 K using a potentiometric measurement system by Dr. Craig Vandegrift of NIST in 1993, who provided these measurements.

d This pertains to the potential probe contacts: the source and drain contacts had zero contact resistance for currents between 0 μA and well over 100 μA.

**Table 2:** Summary of properties of unpassivated samples at 1.1 K

Sample	Plateau centers, width and overlap^a^	*R_x_* = *V_x_*/*I*	Hall plateau value (4*R*_H_/*R*_K_−1)	Breakdown current	Contact resistances
E5	Center: 5.25 T; Widths: 0.06 T to 0.22 T Overlap: ≈ 0.01 T	(0 ± 3) mΩ @ 1.4 K	(0 ± 1) × 10^−6^	250 μA	All but three were 0 Ω; the others were 100 Ω to 500 Ω
E6	Center: 5.05 T Widths: 0 T to 0.2 T Overlap: ≈ 0 T	(0 ± 1) mΩ, except for *V*_35_, *V*_26_, and *V*_46_, which were ≈ (15 ± 6) mΩ^b^	[(0 to 1) ± 1.3] × 10^−6^	300 μA	Three were 0 Ω; others were 100 Ω to 3000 Ω
E7	Center: 5.19 T Widths: 0.15 T to 0.3 T Overlap: ≈ 0.02 T	(0 ± 2) mΩ	[(0 to 3) ± 1] × 10^−6^	250 μA	P4 was 0 Ω; others were 10 Ω to 1500 Ω
E8	Center: 5.27 T Widths: 0 T to 0.2 T Overlap^c^: 0.05 T	*R*_24_,*R*_26_,*R*_46_:[(0 to 1) ± 2] mΩ others^d^: (5 ± 10) mΩ	[(0 to 7) ± 4] × 10^−6^	280 μA	200 Ω to 10000 Ω

a The plateaus in the voltages measured between different probe pairs on the same sample were sometimes centered at slightly different values of the magnetic field, and often different plateaus had different widths. There was, however, a range of magnetic field over which the voltages measured between all probe pairs exhibited plateaus. This range is called the “overlap.”

b The high values were partly due to the fact that the plateaus between different probe pairs did not all occur over the same ranges of magnetic field. For these measurements, the magnetic flux density was set to 5.05 T, at which most of the probe pairs exhibited plateaus or minima. The *V*_26_ and *V*_46_ minima occurred over different ranges of magnetic field which did not contain 5.05 T (See Fig. 4), hence *R_x_* for these two probe pairs appears to be nonzero.

c Two *V_x_* probe pairs exhibited minima, and not well-developed plateaus that were independent of magnetic field over any appreciable range. All other probe pairs exhibited broad plateaus that were about 0.2 T wide and overlapped over a range of 0.05 T centered about 5.27 T.

d The larger noise in the *V_x_* voltages measured on the “odd” side of the sample (*V*_15_, *V*_13_, and *V*_35_) is most probably an artifact of the measurement system, and not reflective of any property of the sample: the “even” side of the device was at the same potential as the source contact on the QHR device, which was connected to the low-voltage terminal of the current source, which was not well isolated from ground. When voltages were measured between probe pairs on the “even” side of the sample, the low-voltage terminals of the DVM and current source coincided; when voltages were measured on the “odd” side of the sample, they did not, giving rise to increased noise in the measurement.

## Footnotes

1 Certain commercial equipment, instruments, or materials are identified in this paper to foster understanding. Such identification does not imply recommendation or endorsement by the National Institute of Standards and Technology, nor does it imply that the materials or equipment identified are necessarily the best available for the purpose.

2 The GaAs is readily and severely oxidized and corroded in water, particularly at elevated temperatures—see Ref. [29].

3 The samples were attached to a glass carrier plate rather than directly to the header, to facilitate removal of the sample from the header without disrupting the wires bonded to the pads on the sample. Wires were bonded between the sample and pads on the carrier plate, so only the bonds between the pads on the carrier plate and the header pins would have to be broken when the sample was removed from the header. Both of these bonds are more easily replaced than the bonds to the pads on the fragile GaAs chip.

4 All solvents used in this work were either “electronic grade” (low concentration of heavy metals and filtered to remove particulate contamination) or “reagent grade.”

5 The “overlap region” of 0.02 T reported in Table 2 includes all probe potentials, including *V*_12_, which occurred over a range in magnetic flux density that was about 0.1 T higher than the other plateaus.

6 This is an agreement with observations made by others. See, for example, Ref. [30].
